# Transcriptome of the *Lymantria dispar* (Gypsy Moth) Larval Midgut in Response to Infection by *Bacillus thuringiensis*

**DOI:** 10.1371/journal.pone.0061190

**Published:** 2013-05-01

**Authors:** Michael E. Sparks, Michael B. Blackburn, Daniel Kuhar, Dawn E. Gundersen-Rindal

**Affiliations:** United States Department of Agriculture -ARS Invasive Insect Biocontrol and Behavior Laboratory, Beltsville, Maryland, United States of America; Swedish University of Agricultural Sciences, Sweden

## Abstract

Transcriptomic profiles of the serious lepidopteran insect pest *Lymantria dispar* (gypsy moth) were characterized in the larval midgut in response to infection by *Bacillus thuringiensis kurstaki*, a biopesticide commonly used for its control. RNA-Seq approaches were used to define a set of 49,613 assembled transcript sequences, of which 838, 1,248 and 3,305 were respectively partitioned into high-, mid- and low-quality tiers on the basis of homology information. Digital gene expression profiles suggested genes differentially expressed at 24 hours post infection, and qRT-PCR analyses were performed for verification. The differentially expressed genes primarily associated with digestive function, including α-amylase, lipase and carboxypeptidase; immune response, including C-type lectin 4; developmental genes such as arylphorin; as well as a variety of binding proteins: cellular retinoic acid binding protein (lipid-binding), insulin-related peptide binding protein (protein-binding) and ovary C/EBPg transcription factor (nucleic acid-binding). This is the first study conducted to specifically investigate gypsy moth response to a bacterial infection challenge using large-scale sequencing technologies, and the results highlight important genes that could be involved in biopesticide resistance development or could serve as targets for biologically-based control mechanisms of this insect pest.

## Introduction

*Lymantria dispar* (Lepidoptera: Erebidae), the gypsy moth, is the most serious insect pest of forest and shade trees in the Northeastern United States. The gypsy moth is a polyphagous insect; North American larval populations feed on over 300 different shrub and tree species [1], including forest, shade, ornamental and fruit trees and shrubs. Gypsy moth larvae have been responsible for defoliating an average of 3.0 million forested acres per year over the past 25 years, as well as trees and shrubs in residential areas, causing significant economic impacts. Great effort has been made to slow the spread, yet attempts to fully contain this insect pest have had variable success and its range has continued to expand into the Midwest and South. Several control measures have been implemented in the U.S. to reduce gypsy moth spread, including stringent quarantine practices, augmentative release of natural enemies (mainly parasitoids), application of chemical pesticides (diflubenzuron =  Dimilin®), use of a chemical pheromone for mating disruption (Disparlure), or ground or aerial application of formulations containing either a specific nucleopolyhedrovirus (Gypchek®) or the gram-positive soil bacterium *Bacillus thuringiensis* (Bt). These microbe-based bioinsecticides have been used with variable success and bioinsecticide resistance has evolved rapidly in gypsy moth larval populations; the reasons for this are currently unknown. Limited genetic information exists for *L. dispar*. The only large publicly available data set for gypsy moth-associated genes was recently generated by characterizing the transcriptome from the *L. dispar*-derived cell line IPLB-Ld652Y [2]; however, this *L. dispar* cell line does not completely reflect the gene and gene systems expressed by whole insect larvae. Comprehensive genetic evaluations are needed to reveal genetic sensitivities of the pest, improve bioinsecticide selection, recognize genes that are determinants of disease and resistance development, and facilitate targeted pest management.

Strains of Bt produce crystalline (Cry) proteins that possess insecticidal activity and act as a gut poison but are harmless to vertebrates and plants [3]. *B. thuringiensis* subsp. *kurstaki*, which produces three lepidopteran-insecticidal Cry1A proteins, is the strain most commonly employed as a bioinsecticide against lepidopteran pest larvae. Once ingested, Cry proteins are proteolytically activated by enzymatic cleavage in the alkaline larval midgut, where they bind to cadherin receptors and undergo insertion into the membrane of epithelial cells [4]. The bound toxin is changed in conformation and then able to bind to additional receptors [5]–[7]. The activated toxins ultimately form pores in epithelial cell membranes causing osmotic lysis and sloughing of damaged cells from the basement membrane of the midgut epithelium [8], [9]. The midgut becomes paralyzed, resulting in cessation of feeding activity and leading to death [10]. A different mechanism of Cry activity was demonstrated in *Trichoplusia ni* where Cry protein binding caused induction of the adenyl cyclase/PKA signaling pathway, leading to significant midgut cytological changes and lysis [11].

Lepidopteran midgut tissues are the site for digestion, secretion, absorption, enzymatic activities, toxin binding and activation, and the center of activity for biopesticide detoxification and resistance development. Several distinct midgut genes have been identified as potentially associated with lepidopteran resistance development to Bt. Candas et al. (2003; [12]) identified changes in numerous midgut epithelial cell proteins in a *Plodia interpunctella* colony exhibiting resistance to Bt, including decreased chymotrypsin, decreased cell adhesion protein peroxinectin, and increased prophenoloxidase. Soberón et al. (2007; [13]) demonstrated that susceptibility to Bt toxin Cry1Ab was reduced by cadherin gene silencing with RNA interference in *Manduca sexta*, and suggested cadherin increases Bt toxicity by facilitating toxin oligomerization. A global transcriptome-based analysis of Bt-resistance development in *Heliothis virescens* by Zhu et al. (2011; [14]) revealed genes potentially related to Bt activation and resistance (proteinases, cadherins, aminopeptidases, and alkaline phosphatases) as well as detoxification (cytochrome P450 oxidases, glutathione S-transferases, esterases, sodium channels, and cytochrome oxidases).

In the current study, a global genetic and transcriptome-based analysis of the gypsy moth midgut in response to infection by *B. thuringiensis kurstaki* was examined using bioinformatics techniques coupled with high throughput next-generation DNA sequencing. An RNA-Seq transcriptome dataset was obtained from synchronized late third instar *L. dispar* larvae midgut tissues, a set of high-quality gypsy moth gene structures was delineated and functionally annotated, and the transcriptome-level response of gypsy moth midgut tissue to infection by Bt was analyzed. Highly differentially expressed transcripts were further validated by quantitative real-time PCR analyses to gain insight into the global larval gypsy moth response to Bt infection and the larval genes that may be involved in resistance mechanisms or that could serve as targets for improved biopesticides.

## Results and Discussion

This study establishes a large collection of high-quality larval *L. dispar* transcript sequences that should be of considerable value to the lepidopteran community in general and to gypsy moth researchers in particular. The sequence analysis protocol described below in Materials and Methods resulted in 838 distinct *L. dispar* gold-tier PUTs associated with 732 distinct NR proteins. Similarly, 1,248 silver-tier PUTs associating with 989 distinct NR proteins, and 3,305 bronze-tier PUTs associating with 2,770 distinct NR proteins, were identified. (One or more PUTs may hit to a single NR protein because, for instance, certain subsets of the PUTs may represent transcript isoforms of the same gene.) Eight hundred (≈92.4%) of the 838 gold-tier gene protein translations exhibited a hit in Pfam-A, yielding 1,059 Pfam terms (565 of which were unique) and 1,470 GO terms (408 of which were unique). 11,094 sequences from the complete PUT collection (≈22.4%) exhibited a longest ORF significantly similar to a Pfam protein family per the aforementioned criteria, of which 3,069 were unique.

**Figure 1:**
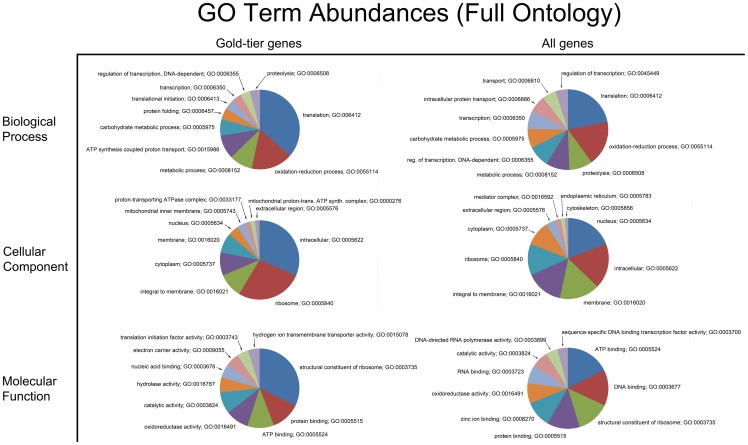
GO term abundances (full ontology). Shown are pie charts presenting the relative abundance of the ten most frequently encountered gene ontology (GO) terms for both the 838 gold-tier PUT subset, as well as the complete 49,613 PUT set. GO terms contribute additional annotation information for inferred gypsy moth genes along three dimensions: biological process, cellular component and molecular function. The GO terms recovered were stratified according to these ontology domains.

**Figure 2:**
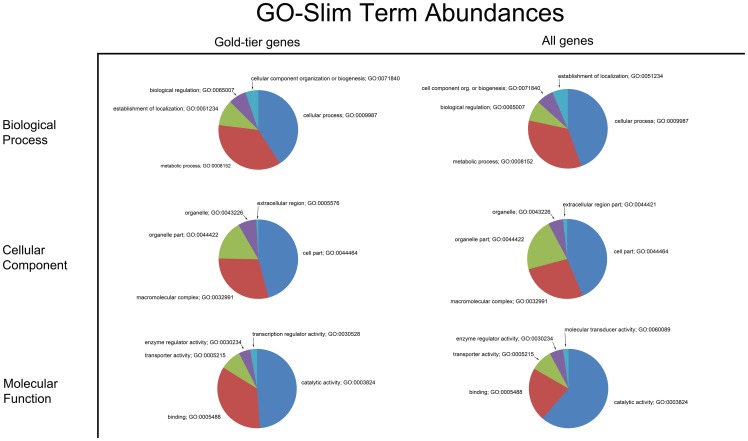
GO-Slim term abundances. Shown are pie charts presenting the relative abundance of the five most frequently encountered GO-Slim terms for both the 838 gold-tier PUT subset, as well as the complete 49,613 PUT set. GO-Slim terms provide a less granular representation of the gene annotation information conveyed by terms from the full GO ontology, thereby offering a higher-level perspective on the functional capacity contained in the *Lymantria dispar* gene set relative to fully-qualified terms. GO-Slim terms are stratified with respect to the ontology's three primary domains: biological process, cellular component and molecular function.

The 25 most abundant Pfam families encountered in the complete PUT dataset is shown in Table 1; those for the high-quality/gold-tier genes are presented in Table S1. 294 GO terms were recovered from the gold-tier gene set, and 1,675 from the comprehensive set of PUTs. (Pfam families can have zero, one, or more associated GO terms). Figure 1 presents the ten most abundant GO terms identified for the high-quality and complete PUT datasets, partitioned into each of the three main GO sub-groupings: Biological Process, Cellular Component and Molecular Function. Figure 2 presents similar information, but using the more abstract GO-Slim terminology. Only GO terms classified under Molecular Function could be mapped to a KEGG entry – for the gold-tier gene set, 7/148 (≈4.7%) of its unique GO terms had an associated KEGG entry, while for the complete PUT collection, this value was 43/492 (≈8.7%). The KEGG entities recovered are exhaustively listed in Table S2, for both gene sets.

**Table 1:** The 25 most abundant Pfam families encountered in the complete gypsy moth PUT collection.

Pfam Hit	Pfam Description	Counts
PF00400.25	WD domain, G-beta repeat	174
PF00069.18	Protein kinase domain	170
PF00096.19	Zinc finger, C2H2 type	149
PF00135.21	Carboxylesterase	138
PF07690.9	Major Facilitator Superfamily	135
PF00089.19	Trypsin	119
PF00076.15	RNA recognition motif. (a.k.a. RRM, RBD, or RNP domain)	106
PF00067.15	Cytochrome P450	100
PF07679.9	Immunoglobulin I-set domain	91
PF00083.17	Sugar (and other) transporter	84
PF00560.26	Leucine Rich Repeat	83
PF01607.17	Chitin binding Peritrophin-A domain	75
PF00106.18	short chain dehydrogenase	74
PF00078.20	Reverse transcriptase (RNA-dependent DNA polymerase)	65
PF00023.23	Ankyrin repeat	63
PF00501.21	AMP-binding enzyme	58
PF00270.22	DEAD/DEAH box helicase	56
PF00041.14	Fibronectin type III domain	52
PF00071.15	Ras family	51
PF00435.14	Spectrin repeat	50
PF00171.15	Aldehyde dehydrogenase family	46
PF00271.24	Helicase conserved C-terminal domain	43
PF07714.10	Protein tyrosine kinase	42
PF00201.11	UDP-glucoronosyl and UDP-glucosyl transferase	42
PF00005.20	ABC transporter	40

Our results are reasonably similar to those of related studies focusing on lepidopteran midgut gene content – including studies on *Manduca sexta* [15] and *Chilo suppressalis* [16] – implying that a core set of functional midgut genes operates across the Lepidoptera. For example, on the basis of NCBI NR comparisons, 4 serpin, 14 lectin, 16 lysozyme, 29 cytochrome P450, 50 glutathione S-transferase, 26 heat shock protein, 19 aminopeptidase, and 14 chymotrypsin-like protease PUTs were encountered in the comprehensive PUT collection, which corresponds well with observations made in these earlier studies. Further, cytochrome P450s, gluthathione S-transferases, and several heat shock proteins have been linked to detoxification and stress adaptation and response in other Lepidopteran systems [17]. Non-tissue-specific surveys of gene content in the Lepidoptera have also been performed for *Plutella xylostella* [18] and *Spodoptera exigua* [19], which also support the notion of a common gene set across this order. Interestingly, two transcript fragments were identified in the bronze-tier PUT subset which appeared to encode a gypsy moth β-fructofuranosidase gene having high similarity to a gene reported in *M. sexta* [15], thereby expanding the incidence of this horizontally-acquired bacterial gene among the Lepidoptera to the *Lymantria* genus.

**Figure 3:**
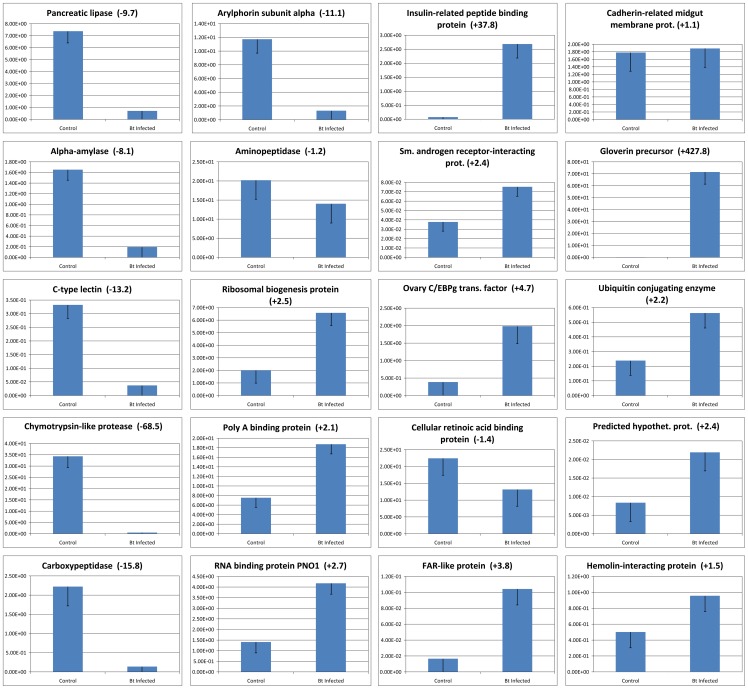
qRT-PCR Results. Quantitative real-time PCR analysis data for 20 select *Lymantria dispar* midgut genes are presented. Three technical replicates were performed for each of three biological replicates. The height of each box represents the mean average of sample-specific 2^-ΔCt^ values, while associated error bars denote the standard error of the mean. Fold changes are shown in parentheses.

In addition to qualitatively defining a set of reliable gypsy moth gene structures, the RNA-Seq data were quantitatively evaluated to suggest which gold-tier genes were expressed differentially. Table 2 displays the 15 gypsy moth genes exhibiting the greatest differences in RNA-Seq-inferred expression levels upon Bt infection, and an exhaustive listing is available in Table S3. Seventy-eight genes exhibited a greater than five-fold change in expression level. A subset of 20 *L. dispar* genes was selected for qRT-PCR validation on the basis of these results, as well as on intuitions concerning the possible biological relevancy of other genes not evaluated by digital expression. The resulting qRT-PCR expression profiles for these selected genes are presented in Figure 3. In general, at 24 hours post infection, transcription and binding-related genes exhibited elevated expression levels, while enzymatic-/digestion-related gene expression was strongly inhibited, as was observed with α-amylase, (pancreatic) lipase, carboxypeptidase and chymotrypsin-like protease. The observation of sharply decreased chymotrypsin-like protease expression corresponds well with proteomic-based data reported for *Plodia interpunctella* [12].

**Table 2:** The 15 gypsy moth genes having the largest digital gene expression level perturbations following *Bacillus thuringiensis* infection.

FoldDiff	Direction	Control	*Bt* midgut	NRgene
166.94	down	4.00E-04	2.40E-06	gb|ABU98614.1| alpha-amylase [Helicoverpa armigera]
103.98	down	8.31E-04	7.99E-06	gb|ABR88238.1| chymotrypsin-like protease C8 [Heliothis virescens]
98.38	down	3.93E-05	3.99E-07	ref|NP_001165397.1| C-type lectin 4 [Bombyx mori]
91.88	down	2.20E-04	2.40E-06	gb|ACB54943.1| lipase [Helicoverpa armigera]
69.57	up	3.39E-07	2.36E-05	ref|XP_396381.2| PREDICTED: similar to tubulin-specific chaperone a [Apis mellifera]
65.62	down	4.98E-04	7.59E-06	gb|ACU00133.1| chymotrypsin-like protein precursor [Spodoptera litura]
52.80	down	8.44E-05	1.60E-06	ref|XP_001662277.1| hypothetical protein AaeL_AAEL012148 [Aedes aegypti]
49.52	up	6.78E-07	3.36E-05	ref|NP_001037102.1| ovary C/EBPg transcription factor [Bombyx mori]
44.85	down	1.07E-03	2.40E-05	gb|ACD37364.1| pancreatic lipase 2 [Mamestra configurata]
42.66	down	6.30E-04	1.48E-05	gb|ACH86113.1| molt-regulating transcription factor HaHR3 [Helicoverpa armigera]
39.63	down	1.74E-04	4.39E-06	gb|ACX53715.1| aldo-keto reductase [Heliothis virescens]
38.12	up	1.02E-06	3.87E-05	ref|XP_001604022.1| PREDICTED: hypothetical protein [Nasonia vitripennis]
25.74	up	2.03E-06	5.23E-05	gb|AAF61949.1|AF236641_1 insulin-related peptide binding protein [Spodoptera
24.91	down	7.96E-05	3.20E-06	gb|AAS82583.1| midgut carboxypeptidase A1 [Trichoplusia ni]
21.22	up	1.02E-06	2.16E-05	ref|NP_001040248.1| glycolipid transfer protein [Bombyx mori]

The FoldDiff column indicates the fold change in expression level; Direction whether the gene was up- or down-regulated; Control and *Bt* midgut provide sample-specific digital expression counts normalized to the total number of reads per sample; and NRgene describes the best homolog identified in the NR protein sequence database for the associated transcript.

Interestingly, the *L. dispar* C-type lectin 4 gene was also highly down-regulated in midgut tissue (-13.2 fold). This gene belongs to a family of calcium-dependent glycoprotein receptors associated with lepidopteran innate immunity [20]; specifically, they bind various cell wall components of invading microorganisms, facilitating nodule formation by hemocytes and subsequent elimination of alien microbial agents via the hemocoel. It would seem that a bacterial infection should promote enhanced, not repressed, levels of immune-related gene products like the gypsy moth C-type lectin 4 gene. The observed down-regulation may indicate that Bt, either directly or indirectly, enhances its entomopathogenicity by means of repressing such immune-related genes as the lectins.

Another important lepidopteran midgut-expressed gene is arylphorin, a hexameric storage protein which has a demonstrated role in lepidopteran midgut stem cell proliferation and differentiation [21], was highly down-regulated in *L. dispar* midgut tissue after Bt infection (-11.1 fold). This was somewhat unexpected given the roles of arylphorin in the midgut. Forcada et al. (1999; [22]) and Martínez-Ramírez et al. (1999; [23]) proposed a Bt resistance mechanism for *Heliothis virescens* based on an increased capacity to repair damage to the midgut. Loeb et al. (2001; [24]) demonstrated in primary midgut cell cultures that transient exposure of midgut cells to Cry1Ac killed differentiated cells, but subsequently stimulated proliferation and differentiation of stem cells. High levels of arylphorin expression have also been implicated in the development of Bt resistance in *Spodoptera exigua* [25]. Monomeric subunits of arylphorin were shown to stimulate proliferation of *M. sexta* midgut stem cells *in vitro* [26]. The reduced expression of arylphorin in the *L. dispar* midgut at 24 hours post infection may suggest a generalized response of midgut cells to slough without (yet) stimulating new cells.

Genes related to anti-bacterial or anti-pathogen response in *L. dispar* and other lepidopterans are of high interest and several were up-regulated, among them hemolin-interacting protein and a gloverin precursor, the latter of which was the most abundantly up-regulated gene surveyed in our qRT-PCR panel (+427 fold). The gloverin peptide was originally described from the giant silk moth *Hyalophora gloveri* [27] as anti-microbial and highly induced in midgut tissue following exposure to Bt. As with many antimicrobial peptides, the transcriptional regulation of gloverin is regulated by interaction with the spätzle-Toll receptor [28]. In *Spodoptera exigua*, RNAi knockdown of gloverin expression caused developmental retardation and increased susceptibility to Bt [29]. Gloverin expressed in *Trichoplusia ni* was shown to increase in response to infection with *Autographa californica* M nucleopolyhedrovirus, though it remains unclear whether this gene may also have anti-viral properties or its increased expression is coincidental [30].

Another interesting gene highly up-regulated in *L. dispar* midgut after Bt infection is an insulin-related peptide-binding protein (+37.8 fold). This is a putative endocrine factor that may be important in the lepidopteran insulin-signal pathway and immune response, and a similar protein from *Bombyx mori* (BmIBP2) was recently shown to be up-regulated on infection with cytoplasmic polyhedrosis virus (BmCPV) in midgut but not other tissues [31]. Additional endocrine-associated genes are of interest, including the ovary C/EBP transcription factor up-regulated in Bt-infected gypsy moth midgut (+4.7 fold). In mammalian skin cells these factors are known to regulate wound repair and EGF receptor signaling [32], which suggests a potential role in host defense.

The FAR-like gene was also up-regulated in gypsy moth midgut (+3.8). Fatty acyl reductase (FAR) genes are important in the biosynthesis of insect pheromone as shown for the pheromone gland-specific fatty-acyl reductase of the lepidopteran adzuki bean borer, *Ostrinia scapulalis* [33]. FAR and FAR-like genes may heavily influence the pheromone composition in *Lepitoptera* and contribute to their pheromone specificity [34]. This gene has been shown to be up-regulated in *B. mori* at a later developmental stage and prior to eclosion, and could perhaps have functional flexibility, as described by Liénard and Löfstedt (2010; [35]), in promoting phenotypic transitions in the pheromone signaling pathway that is not yet active in the larval gypsy moth; perhaps this gene has functional plasticity which needs to be explored.

Several differentially-regulated genes of unknown function may have significant roles in the lepidopteran response to Bt infection. For instance, a number of hypothetical proteins were up-regulated in the present study; one of these, presented in Fig. 3, exhibited limited similarity (53% identity) to a fungal protein involved in the transport of fructose-1,6-bisphosphatase from vesicles to vacuoles for degradation [36]. The observation of a concomitant increase in ubiquitin conjugating enzyme, a gene known to play a key role in protein degradation [37], suggests that Bt infection may elicit an increase in proteolytic activities.

The gene sets identified in this study will be of utility to entomologists studying the functional capacity inherent in the *L. dispar* gene repertoire, and in teasing apart its molecular evolutionary relationships with other lepidopteran species. The quantitative results highlight a variety of larval-stage *L. dispar* genes that respond within 24 hours to infection by *Bacillus thuringiensis*. Bt-responsive midgut genes have been discussed in other studies of lepidopteran pests, such as the beet armyworm [25] and tobacco budworm [14], and our similar findings support the shared core set of responsive midgut genes operating across this order (e.g., lectin, cytochrome p450, amylase, chymotrypsin). Interestingly, the general pattern of down-regulation in metabolism-associated genes, and a mixed response among immune-related genes, corresponds very well to observations recently made of a Coleopteran species, *Tenebrio molitor*, following ingestion of Cry3Aa protoxin [38]. The responses observed for genes such as arylphorin and the FAR-like transcript provide opportunities for further exploring the mechanisms underlying Bt entomopathogenicity and its associated host response. Likewise, genes induced in the infected midgut by multiple classes of pathogens (e.g., insulin-related peptide binding protein and gloverin) represent key lepidopteran genes for further assessment. If they prove to be sufficiently species-specific, these genes could suggest possible targets for RNAi-mediated gene disruption to be used in ongoing biocontrol efforts slowing the spread of this destructive insect pest.

## Materials and Methods

### Insect Rearing and Dissection

*L. dispar* egg masses were obtained from the USDA APHIS rearing facility, Otis AFB, Massachusetts. Larvae were hatched and reared on high wheat germ artificial diet [39] in 180-ml plastic cups with paper lids under conditions of 24±1°C, RH 55–60%, L16:D8. All *L. dispar* larvae used for this study were reared from a single egg mass to reduce variability. Larvae were staged by rearing in groups of approximately 50 individuals in 180-ml plastic cups until head capsule formation was observed signifying entry into the larval moult to 3rd instar. Larvae which entered the third instar at the same time were then reared with 10-15 larvae per plastic cup for 48 h. The synchronous larvae were starved for 24 hrs then orally inoculated by feeding a 3×3 mm diet block containing 10^8^ *B. thuringiensis kurstaki* spores, obtained from Thuricide®, per ml diet [40], or diet alone for control larvae.

### RNA Extraction

Midgut tissues (containing anterior, middle, and posterior regions as well as the peritrophic matrix) from a total of 31 control uninfected and 28 Bt-infected larvae, respectively, were dissected from the larvae at a time point 24 hours post infection, rinsed thoroughly in 1xPBS to remove debris, and pooled. Midgut tissue RNAs were extracted immediately upon dissection and used to generate RNA-seq libraries. RNAs were stored frozen at −80°C. Additional control and Bt-infected midguts were stored in RNAlater™ (Ambion/Life Technologies, Carlsbad, CA) until RNAs were extracted to obtain biological replicates for qRT-PCR. Total RNA for RNA-seq libraries was extracted using the mirVana miRNA Isolation Kit (Ambion/Life Technologies) according to the manufacturer's protocol for total RNA isolation, with homogenization of the midgut tissue performed on a FastPrep 24 homogenizer (MP Biomedicals, Santa Ana, CA).

### RNA-seq libraries

RNA quantity and quality was determined using an Agilent RNA 6000 Nano kit on a 2100 Bioanalyzer system (Agilent Technologies, Santa Clara, CA) according to the manufacturer's instructions. Samples for sequencing of mRNA were prepared using the mRNA-Seq Sample Prep Kit (Illumina, San Diego CA) as per the manufacturer's protocol. Library quantity and quality was assessed using an Agilent DNA 1000 kit on a 2100 Bioanalyzer, and these materials were then sequenced using an Illumina GAII instrument (Illumina).

### RNA-Seq Data

94,477,033 72 bp reads were generated, available in the NCBI sequence read archive under accession no. SRA058966. Illumina quality scores were transformed to the Phred scoring scale [41], and read sets were cleaned using the FASTX-toolkit (http://hannonlab.cshl.edu/fastx_toolkit): Artifact reads were purged, and terminal spans of bases having Phred scores not more than 20 (corresponding to a 1/100 error rate) were clipped – resultant reads were required to be at least 36 bases long. At least 90% of bases in a read were required to exhibit a Phred score of 21 or higher. Of the remaining reads, all bases having quality scores of 20 or less were masked with the symbol ‘N’. 3,061,431,248 bases were retained from 6,802,346,376 originally sequenced (45% retention). These were aligned against a fruit fly rRNA gene locus – accession no. M21017, comprising 2S, 5.8S, 18S and 28S rRNA sequences[42] – using the Blat program with default parameters [43]. 7,122,506 reads containing 498,351,320 usable bases were retained for transcript assembly and digital gene expression characterization. Table 3 presents some descriptive statistics of these data.

**Table 3:** Impacts of quality filtering and rRNA depletion on resultant RNA-Seq dataset sizes.

		Untreated Midgut Tissue				*Bt*-infected Midgut Tissue			Independent Study, Midgut Tissue		
		1	2	3	Total	4	5	Total	6	7	Total
Raw Data	Bases	905,299,920	1,156,410,792	777,226,032	2,838,936,744	953,698,248	1,059,143,040	2,012,841,288	857,218,032	1,093,350,312	1,950,568,344
	Reads	12,573,610	16,061,261	10,794,806	39,429,677	13,245,809	14,710,320	27,956,129	11,905,806	15,185,421	27,091,227
Quality Control	Bases	550,084,462	368,559,364	366,374,029	1,285,017,855	518,769,000	458,765,055	977,534,055	433,011,464	365,867,874	798,879,338
	Reads	7,803,203	5,455,352	5,300,524	18,559,079	7,424,324	6,625,479	14,049,803	6,263,601	5,412,992	11,676,593
rRNA Depletion	Bases	106,726,806	38,345,093	61,164,946	206,236,845	106,625,974	69,809,845	176,435,819	75,353,135	40,325,521	115,678,656
	Reads	1,507,602	563,277	880,922	2,951,801	1,507,789	995,710	2,503,499	1,076,817	590,389	1,667,206

### Sequence Analysis

To create a highly-reliable set of gypsy moth genes (“gold tier” – see below), all RNA-Seq samples were pooled and globally assembled using the Velvet/Oases short read assembler suite [44] with its hash length parameter set to 23. This generated a set of 49,613 putatively unique transcripts (PUTs). The assembly exhibited an N50 of 353 bp, with 41,352; 11,798 and 6,362 PUTs being not less than 100; 300 and 450 bp in length, respectively. Known Dipteran repetitive elements were purged using RepeatMasker [45], and the residual PUTs were aligned to the NCBI NR database using Blastx with default parameter settings [46]. Blastx results were parsed under varying levels of stringency to establish three distinct tiers of homology-inferred gene accuracy quality: gold, silver and bronze.

The gold-tier gene set was defined per the following criteria. A Blastx hit had to consist of a single high-scoring segment pair (HSP), so as to minimize consideration of PUTs having potentially significant internal mis-assemblies; a PUT had to be at least 300 bases in length; the alignment's subject sequence (i.e., an NR protein) had to be at least 100 amino acid residues in length; at least 75% of aligned residues had to be positively similar; and the ratio of hit length to subject sequence length had to be at least 90%. Only the top-scoring hit per PUT was considered. End sequences from PUTs that were not incorporated into Blastx-derived alignments were trimmed and resultant CDSs were translated into high-quality gypsy moth protein sequences using the EMBOSS package's Transeq utility [47]. PUTs of the silver-tier gene class had to be at least 100 nucleotides in length and exhibit a hit length covering at least 75% of the NR subject sequence's length; PUTs of the bronze-tier class were required to be at least 100 nucleotides long and have a hit covering at least 30% of the NR protein's length. These represent mutually disjoint sets in that a gold-tier PUT is not multiply listed among those in the silver- or bronze-tier, and silver-tier PUTs are likewise not listed at the bronze level.

### Functional annotation of genes

Gold-tier protein sequences were analyzed against Pfam-A – the manually curated subset of Pfam [48] – using hmmscan [49] with an E-value cutoff of 1E-2. The top hit for the PUT (provided any satisfied the hmmscan inclusion threshold criteria) was selected and used to annotate the gypsy moth protein. In addition, the complete set of PUTs was also assessed for its protein family composition profile: Each assembled transcript was translated in six frames using Transeq, and the longest ORF that resulted in this set was selected for querying Pfam-A. (Where two or more longest ORFs for a given PUT occurred, one was selected arbitrarily.) To provide additional functional information, Pfam families encountered in these datasets were then mapped to Gene Ontology (GO) terms using the January 10, 2011 version of the pfam2go table [50].

To provide a yet more abstract representation of the functional capacity implicit in the gypsy moth transcriptome, each (fine-grained) term's penultimate ancestor was retrieved by traversing the GO directed acyclic graph using the “is_a” relationships embedded in the ontology definition – this was repeated for each of the three main categories of GO terms. Penultimate ancestors, herein referred to as “GO-Slim terms,” only were considered because ultimate ancestors would resolve to one of the three main categorizations, and would be uninformative. Version 1.9 of the kegg2go table was obtained from the Gene Ontology web site (http://www.geneontology.org) and used to map fine-grained GO terms onto Reaction records from the KEGG Ligand database [51].

### Sample-specific gene expression quantification

To identify any putatively up- or down-regulated genes among the gold-tier gene set described above, the unassembled read data were pooled into two groups: uninfected *L. dispar* control (2,951,801 reads) and *B. thuringiensis*- infected *L. dispar* (2,503,499; see Table 3). These were aligned to trimmed gold-tier PUT sequences using Blat. A read was considered as emanating from a particular PUT if and only if at least 95% of its length aligned with perfect sequence identity. A read was associated with at most one PUT, being that having the highest-quality Blat alignment – where multiple such best hits occurred, one among the competing PUTs was arbitrarily selected. Counts emanating from multiple PUTs associated with the same NR protein were accumulated to that protein. Digital expression counts were normalized relative to the total number of reads pooled within the sample.

### Quantitative real-time PCR (qRT-PCR) primer design and validation of *L. dispar* larval midgut gene expression

qRT-PCR analysis was conducted to validate key midgut genes of interest, which were identified either by their exhibition of relatively large expression fold-changes from digital expression analyses, or by intuition about potential gene functions. PrimerPlex 2.61 (PREMIER Biosoft, Palo Alto, CA) was used to design primers for SYBR-Green experiments using PUT template sequences (see Table S4 for primers used). qRT-PCR was conducted using three biological samples: the first replicate utilized the RNA from which the RNA-Seq library was produced, as well as two additional biological replicates that were obtained in the same infection experiment and stored in RNALater. An ABI 7500 Real Time PCR System (Applied Biosystems, Carlsbad CA) was used. For each replicate, first strand cDNA was synthesized from 1–5 ug RNA using Superscript Reverse Transcriptase II (Invitrogen/Life Technologies, Carlsbad, CA). Each qPCR reaction consisted of 6.25 ul of Power SYBR Green PCR Master Mix (Applied Biosystems/Life Technologies, Carlsbad, CA), 50 ng of diluted cDNA and 1 uM of each primer in a total volume of 12.5 ul. Reactions were performed in triplicate to ensure consistent technical replication and run in 96-well plates under the following conditions: 50°C for 2 min, 95°C for 10 min, and 40 cycles of 95°C for 15 sec and 60°C for 1 min. Melting curves (60°C to 95°C) were derived for each reaction to ensure a single product. Relative gene expression was evaluated with DataAssist Software version 3.0 (Applied Biosystems/Life Technologies), using *L. dispar* 18 s rRNA and the elongation factor-Tu gene as endogenous controls for RNA load and gene expression in analyses.

## Supporting Information

Table S1 (DOCX)

Table S2 (DOCX)

Table S3 (XLSX)

Table S4 (XLSX)
