# Supplementary material for: Transcriptome of the Lymantria dispar (Gypsy Moth) Larval Midgut in Response to Infection by Bacillus thuringiensis
Source: PLoS One. 2013 May 1;8(5):e61190. doi: 10.1371/journal.pone.0061190 (PMC3641027; doi:10.1371/journal.pone.0061190)
Supplement: Table S1 — (DOCX) [file pone.0061190.s001.docx]

Supplementary Table 1. The 25 most abundant Pfam families encountered in the high-quality (gold-tier) gypsy moth gene set.

| Pfam Hit | Pfam Description | Counts |
| --- | --- | --- |
| PF00071.15 | Ras family | 16 |
| PF00091.18 | Tubulin/FtsZ family, GTPase domain | 12 |
| PF02798.13 | Glutathione S-transferase, N-terminal domain | 11 |
| PF00227.19 | Proteasome subunit | 11 |
| PF00036.25 | EF hand | 11 |
| PF00076.15 | RNA recognition motif. (a.k.a. RRM, RBD, or RNP domain) | 10 |
| PF00089.19 | Trypsin | 9 |
| PF00179.19 | Ubiquitin-conjugating enzyme | 8 |
| PF01433.13 | Peptidase family M1 | 7 |
| PF00307.24 | Calponin homology (CH) domain | 7 |
| PF00248.14 | Aldo/keto reductase family | 7 |
| PF00106.18 | short chain dehydrogenase | 7 |
| PF00025.14 | ADP-ribosylation factor family | 7 |
| PF00335.13 | Tetraspanin family | 6 |
| PF00270.22 | DEAD/DEAH box helicase | 6 |
| PF00246.17 | Zinc carboxypeptidase | 6 |
| PF00006.18 | ATP synthase alpha/beta family, nucleotide-binding domain | 6 |
| PF07690.9 | Major Facilitator Superfamily | 5 |
| PF02221.8 | ML domain | 5 |
| PF01576.12 | Myosin tail | 5 |
| PF00400.25 | WD domain, G-beta repeat | 5 |
| PF00261.13 | Tropomyosin | 5 |
| PF00180.13 | Isocitrate/isopropylmalate dehydrogenase | 5 |
| PF00171.15 | Aldehyde dehydrogenase family | 5 |
| PF00151.12 | Lipase | 5 |
