# Supplementary material for: Transcriptome of the Lymantria dispar (Gypsy Moth) Larval Midgut in Response to Infection by Bacillus thuringiensis
Source: PLoS One. 2013 May 1;8(5):e61190. doi: 10.1371/journal.pone.0061190 (PMC3641027; doi:10.1371/journal.pone.0061190)
Supplement: Table S2 — (DOCX) [file pone.0061190.s002.docx]

Supplementary Table 2. KEGG Reaction entities recovered from the high-quality gene and PUT datasets using kegg2go.

| Putative Unique Transcripts (PUTs) | |
| --- | --- |
| R03876 | Ubiquitin:protein-lysine N -ligase (AMP-forming) |
| R00955 | UDP-glucose:alpha-D-galactose-1-phosphate uridylyltransferase |
| R04144 | 5-Phospho-D-ribosylamine:glycine ligase (ADP-forming) |
| R01978 | acetyl-CoA:acetoacetyl-CoA C-acetyltransferase |
| R01011 | ATP:glycerone phosphotransferase |
| R00497 | gamma-L-glutamyl-L-cysteine:glycine ligase (ADP-forming) |
| R00253 | L-Glutamate:ammonia ligase (ADP-forming) |
| R03362 | ATP:1-phosphatidyl-1D-myo-inositol 3-phosphotransferase |
| R03665 | L-Valine:tRNAVal ligase (AMP-forming) |
| R01015 | D-glyceraldehyde-3-phosphate aldose-ketose-isomerase |
| R02101 | 5,10-Methylenetetrahydrofolate:dUMP C-methyltransferase |
| R03738 | 3-alpha(S)-Strictosidine tryptamine-lyase |
| R01529 | D-Ribulose-5-phosphate 3-epimerase |
| R01056 | D-ribose-5-phosphate aldose-ketose-isomerase |
| R00549 | ATP:riboflavin 5'-phosphotransferase |
| R04496 | S-Adenosyl-L-methionine:protein-C-terminal-S-farnesyl-L-cysteine |
| R00036 | 5-aminolevulinate hydro-lyase (adding 5-aminolevulinate and |
| R04591 | 1-(5-Phosphoribosyl)-5-amino-4-carboxyimidazole:L-aspartate ligase |
| R01818 | D-Mannose 6-phosphate 1,6-phosphomutase |
| R01512 | ATP:3-phospho-D-glycerate 1-phosphotransferase |
| R02055 | Phsophatidyl-L-serine carboxy-lyase |
| R00965 | orotidine-5'-phosphate carboxy-lyase (UMP-forming) |
| R01724 | Nicotinate D-ribonucleotide:diphosphate phosphoribosyltransferase |
| R00112 | NADPH:NAD+ oxidoreductase |
| R00104 | ATP:NAD+ 2'-phosphotransferase |
| R03433 | ATP:1D-myo-inositol-1,4,5-trisphosphate 3-phosphotransferase |
| R01184 | myo-Inositol:oxygen oxidoreductase |
| R07324 | 1D-myo-inositol-3-phosphate lyase (isomerizing) |
| R00004 | diphosphate phosphohydrolase; |
| R02082 | (R)-Mevalonate:NADP+ oxidoreductase (CoA acylating) |
| R00894 | L-glutamate:L-cysteine gamma-ligase (ADP-forming) |
| R01364 | 4-fumarylacetoacetate fumarylhydrolase |
| R00310 | protoheme ferro-lyase (protoporphyrin-forming) |
| R01868 | (S)-dihydroorotate:quinone oxidoreductase |
| R00130 | ATP:dephospho-CoA 3'-phosphotransferase |
| R00893 | L-Cysteine:oxygen oxidoreductase |
| R02467 | Cysteamine:oxygen oxidoreductase |
| R03220 | Coproporphyrinogen:oxygen oxidoreductase(decarboxylating) |
| R01135 | IMP:L-aspartate ligase (GDP-forming) |
| R00178 | S-adenosyl-L-methionine carboxy-lyase |
| R02732 | ATP:D-fructose-6-phosphate 2-phosphotransferase |
| R02301 | 5-Formyltetrahydrofolate cyclo-ligase (ADP-forming) |
| R04734 | 4a-hydroxytetrahydrobiopterin hydro-lyase |
|  |  |
| Hiqh-Quality Genes | |
| R01015 | D-glyceraldehyde-3-phosphate aldose-ketose-isomerase |
| R02101 | 5,10-Methylenetetrahydrofolate:dUMP C-methyltransferase |
| R01529 | D-Ribulose-5-phosphate 3-epimerase |
| R04591 | 1-(5-Phosphoribosyl)-5-amino-4-carboxyimidazole:L-aspartate ligase |
| R01512 | ATP:3-phospho-D-glycerate 1-phosphotransferase |
| R01184 | myo-Inositol:oxygen oxidoreductase |
| R03220 | Coproporphyrinogen:oxygen oxidoreductase(decarboxylating) |
